# Tislelizumab plus AG regimen for long-term CR in 2 patients with metastatic BRCA-mutation pancreatic cancer: A case report

**DOI:** 10.1097/MD.0000000000040997

**Published:** 2024-12-27

**Authors:** Feng Gao, Xue Xu, Chunhong Chen, Lili Lv

**Affiliations:** a Department of Oncology, Beidahuang Group General Hospital, Harbin, China.

**Keywords:** AG regimen, BRCA mutations, case report, olaparib, pancreatic cancer, tislelizumab

## Abstract

**Rationale:**

An increased risk of pancreatic cancer (PC) is associated with breast cancer susceptibility gene (BRCA) mutations. Given limited therapies, there remains an urgent need to explore novel treatment strategies.

**Patient concerns:**

We report 2 metastatic PC patients with BRCA mutations who initially did not respond to standard therapies.

**Diagnoses:**

Case 1 was diagnosed with metastatic PC harboring a BRCA1 mutation, and case 2 was diagnosed with metastatic PC harboring a BRCA2 mutation.

**Interventions:**

Both patients received a modified AG regimen (albumin-bound paclitaxel plus oxaliplatin) combined with tislelizumab, followed by maintenance therapy with olaparib plus tislelizumab after 6 cycles.

**Outcomes:**

Both patients achieved complete response, with complete response lasting for 19 months in case 1 and 18 months in case 2.

**Lessons:**

The combination of triple therapy followed by maintenance therapy shows promise as a potential treatment option for PC patients with BRCA mutations. Further clinical investigation is needed to confirm its efficacy and safety.

## 1. Introduction

Pancreatic cancer (PC) is a fatal malignancy.^[1]^ Most patients are diagnosed with metastatic or advanced diseases^[2]^ and have a poor prognosis with a 5-year overall survival (OS) rate of 2% and a median life expectancy of <1 year.^[3]^ Among these, 4% to 7% of patients with PC exhibit breast cancer susceptibility gene (BRCA) mutations.^[4]^ Although the AG regimen (albumin-bound paclitaxel plus gemcitabine) is the first-line treatment for PC patients, the therapeutic effect is far from satisfactory, with a median OS of <1 year.^[5]^ Hence, there remains an urgent need to provide a novel regimen or optimize the existing therapy to achieve better efficacy.

It was previously documented that PC with BRCA mutations exhibited increased sensitivity to platinum chemotherapy due to impaired homologous recombination,^[5]^ potentially further resulting in the death of tumor cells. Hence, we modified the regimen, substituting gemcitabine with platinum (defined as a modified AG regimen), with the expectation of achieving enhanced efficacy. Besides, the fact that chemotherapy may promote immunogenic cell death in PC^[6]^ and BRCA-mutant PC is frequently related to an increased tumor mutation burden and PD-1 expression,^[7]^ forms the basis of an anti-PD-(L)1 agent combined with chemotherapy, as evidenced by a promising survival (mOS, 15 months) in a chemotherapy plus immunotherapy trial.^[8]^ Tislelizumab, an anti-PD-1 monoclonal antibody, has made great progress in cancer treatment in recent decades. Together, we reasoned that tislelizumab may exhibit synergistic effects when combined with a modified AG regimen, while data on this combination are limited.

Furthermore, the optimization of maintenance therapy may also partly contribute to efficacy in BRCA-mutant PC patients. The poly (ADP-ribose) polymerase (PARP) inhibitor olaparib as a maintenance therapy has shown clinical efficacy (significantly improved progression-free survival) in BRCA-mutant PC, as previously demonstrated in a phase III POLO trial.^[4]^ Despite the success of olaparib, the limited maintenance therapies prompted us to expand treatment options, which was crucial for BRCA-mutant PC. The maintenance therapy of olaparib combined with tislelizumab may be a promising option.

Here, we presented the clinical histories of 2 patients, the reason for selecting the triple therapy followed by maintenance therapy, and treatment responses. This report showed the potential benefits of the triple therapy followed by maintenance therapy in PC patients with BRCA mutations, with encouraging efficacy and an acceptable safety profile.

## 2. Case presentations

### 2.1. Case 1

**Figure 1.**
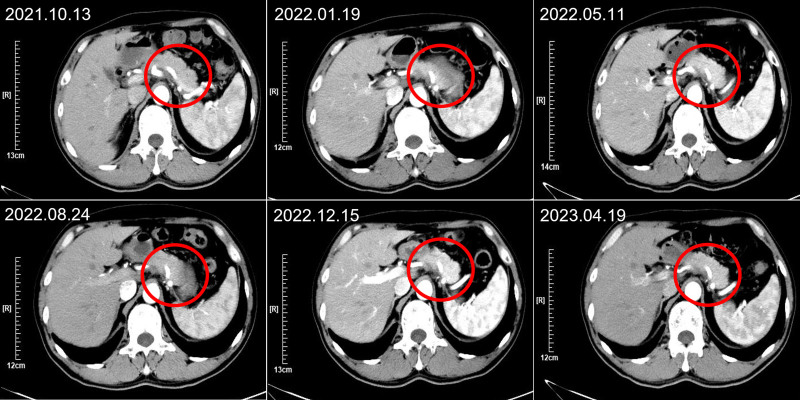
The representative CT images of case 1 after treatment. CT = computed tomography.

**Figure 2.**
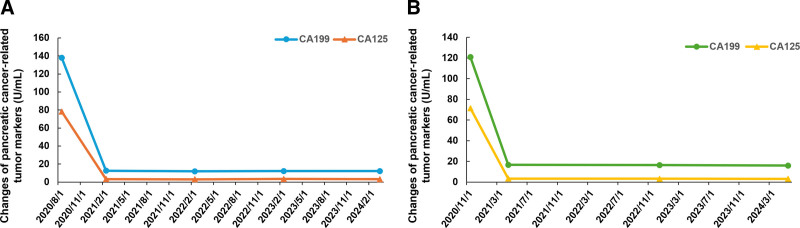
(A) Changes of pancreatic cancer-related tumor markers in case 1. (B) Changes of pancreatic cancer-related tumor markers in case 2. CA199 = carbohydrate antigen 199, CA125 = carbohydrate antigen 125.

A 56-year-old male who had a history of intermittent upper abdominal discomfort for half a year was admitted to the hospital in July 2020. A computed tomography (CT) of the whole abdomen showed a space-occupying lesion between the upper edge of the pancreatic body and tail and the stomach, and multiple retroperitoneal nodules; and lymph node metastasis were considered. Tumor markers carbohydrate antigen 199 (CA199) and carbohydrate antigen 125 (CA125) were 138 and 78.3 U/mL, respectively. Positron emission tomography-CT further demonstrated an irregular soft tissue mass near the lesser curvature of the stomach below the cardia, and the unclear boundary between the lesion and the adjacent gastric wall and pancreatic body and tail; a malignant lesion was considered. The result of an endoscopic ultrasound-guided fine-needle aspiration biopsy exhibited that the patient was diagnosed with moderately to poorly differentiated adenocarcinoma of lymph nodes; combined with immunohistochemistry, it was confirmed to be derived from the pancreas. In conclusion, the clinical diagnosis indicated that the patient had adenocarcinoma of the pancreatic body and tail with abdominal lymph node metastasis. Subsequently, the patient received 3 cycles of AG regimen (albumin-bound paclitaxel 400 mg on day 1 and gemcitabine 1400 mg on day 1 and day 8) plus tislelizumab 200 mg on day 1 every 3 weeks on August 4, 2020. After 3-cycle treatment, the Eastern Cooperative Oncology Group score and Numeric Rating Scale score of this case were 1 and 3, respectively, and the efficacy evaluation was not improved. Following genetic testing showing a mutation of BRCA1, thus, the treatment was changed to a modified AG regimen (albumin-bound paclitaxel 400 mg on day 1 and oxaliplatin 200 mg on day 2) plus tislelizumab 200 mg on day 1 every 3 weeks. After 3 cycles of modified AG regimen, olaparib 300 mg twice a day combined with tislelizumab 200 mg on day 1 every 3 weeks as maintenance therapy was given till now. This patient has remained a CR for 39 months till May 12, 2024 (Fig. 1). The levels of CA199 and CA125 were 12.56 and 3.32 U/mL, respectively, at the initial efficacy evaluation when a CR was achieved (Fig. 2).

### 2.2. Case 2

**Figure 3.**
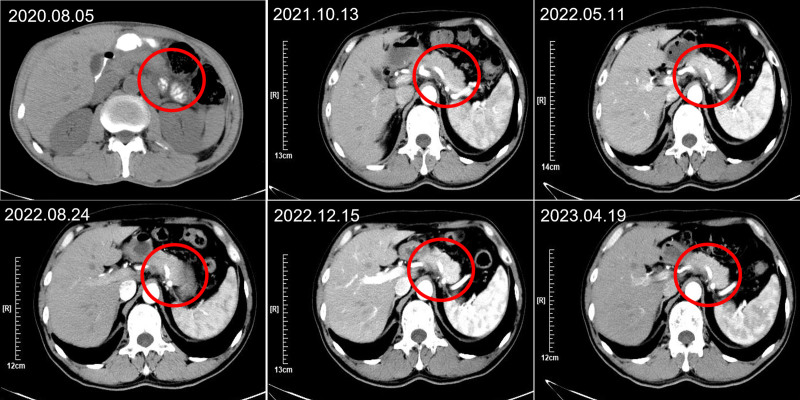
The representative CT images of case 2 after treatment. CT = computed tomography.

A 56-year-old female presented to our hospital with abdominal pain in November 2020. The patient’s family history included bone tumors in her mother (details were unknown), cholangiocarcinoma in her second sister, breast cancer in her seventh sister and second sister’s daughter, and pancreatic cancer in her fifth sister. The examination showed that the Eastern Cooperative Oncology Group score was 1 and the Numeric Rating Scale score was 0; CA199 and CA125 were 121 and 71.5 U/mL, respectively. Positron emission tomography-CT showed a space-occupying lesion in the tail of the pancreas, which was considered malignant lesion; multiple low-density nodules in the liver were considered metastatic lesions; a small nodule shadow in the left upper abdominal cavity (tail of the pancreas) was considered a metastatic lymph node lesion. The pathological diagnosis showed poorly differentiated adenocarcinoma it was considered to be derived from the pancreas. The admission diagnosis revealed that the patient had pancreatic cancer, liver metastases, and cervical cancer after surgery. Subsequently, the patient received AG chemotherapy (albumin-bound paclitaxel 200 mg on day 1 and day 8 and gemcitabine 1200 mg on day 1 and day 8) combined with tislelizumab 200 mg on day 1 every 3 weeks on November 27, 2020. Genetic testing revealed a BRCA2 mutation, and the second cycle of therapy was changed to modified AG regimen (albumin-bound paclitaxel 400 mg on day 1 and oxaliplatin 200 mg on day 2) plus tislelizumab 200 mg on day 1 every 3 weeks. Olaparib 300 mg twice a day combined with tislelizumab 200 mg on day 1 every 3 weeks was maintained after 6 cycles of treatment. Till April 28, 2024, the CR has been maintained for 36 months (Fig. 3). When a CR was achieved, the levels of CA199 and CA125 were 16.60 and 3.40 U/mL, respectively, and remained at low levels after maintenance therapy (Fig. 2).

## 3. Discussion

Our results proposed a potential regimen that the triple therapy (modified AG regimen plus tislelizumab) followed by maintenance therapy (olaparib combined with tislelizumab) for PC patients with BRCA mutations. After using this regimen therapy, both patients maintained a CR, for 39 months in case 1 and 36 months in case 2. The PC tumor markers CA199 and CA125 significantly decreased after triplet therapy and remained at low levels during maintenance therapy.

The increasing evidence supported superior outcomes (of the 10 patients who received platinum-based therapy, a PR was noted in 7 patients and stable disease in 1 patient as the best response^[9]^) of platinum-based therapy in PC patients with BRCA1/2 mutations compared with those in unselected cohorts.^[10]^ Therefore, we applied the modified AG chemotherapy (albumin-bound paclitaxel and oxaliplatin) plus tislelizumab after having no efficacy with the AG chemotherapy (albumin-bound paclitaxel plus gemcitabine) plus tislelizumab in 2 patients, and the symptoms were rapidly relieved, the CR was evaluated after a review of the primary focus and metastatic lymph nodes. NCCN Clinical Practice Guidelines for PC (v1.2023) showed that patients with BRCA1/2 mutations and metastatic diseases, as well as no progression after 4 to 6 months of platinum-based chemotherapy, were recommended for maintenance therapy.^[11]^ Importantly, PARP inhibitors may be a better option of maintenance therapy for BRCA1/2 mutations PC, as indicated previously that patients with BRCA1/2 mutations are more sensitive to DNA-damaging drugs.^[12]^ A phase III POLO trial of the PARP inhibitor olaparib as maintenance therapy for metastatic BRCA-mutation PC also demonstrated favorable efficacy with the median progression-free survival being 7.4 months.^[4]^ Thus, additional potential consideration for promising efficacy was the application of maintenance treatment with olaparib plus tislelizumab in our study. It should be also noted that tumor load mutation in both patients may also partly contribute to the encouraging efficacy of tislelizumab-based regimen in this study. As reported previously, tumor cells with higher levels of tumor load mutation are more easily recognized by the immune system and therefore have a stronger immune response to immune checkpoint inhibitors.^[13]^ Together, we recommend tislelizumab combined with modified AG chemotherapy followed by maintenance therapy with olaparib plus tislelizumab in PC patients with BRCA mutations for long-term clinical benefits. Meanwhile, the observation implies that PC with BRCA mutations is independent of other PCs. Future categorization might consider listing PC with BRCA gene mutation separately, aiming to enhance targeted clinical interventions.

Our study is limited by the small sample size; thus, a large-scale future study is needed to verify the feasibility of this regimen for BRCA-mutant PC patients.

## 4. Conclusions

We have provided a reference for the use of triple therapy consisting of a modified AG regimen in the treatment of PC patients with BRCA gene mutations, and the 2 patients who achieved long-term CR on maintenance therapy have been reported, which also demonstrates that olaparib combined with immunotherapy is an effective maintenance therapy. Besides, these 2 cases suggest that PC with BRCA mutations exhibits distinctive biological characteristics, treatment, and prognosis compared to other PCs, indicating it is an independent pancreatic tumor.

## Author contributions

**Conceptualization:** Feng Gao.

**Methodology:** Feng Gao.

**Writing—original draft:** Feng Gao, Xue Xu.

**Writing—review & editing:** Feng Gao, Xue Xu, Chunhong Chen, Lili Lv.

**Data curation:** Xue Xu.

**Formal analysis:** Xue Xu.

**Resources:** Chunhong Chen, Lili Lv.
